# Avoidant decision making in social anxiety: the interaction of angry faces and emotional responses

**DOI:** 10.3389/fpsyg.2014.01050

**Published:** 2014-09-29

**Authors:** Andre Pittig, Mirko Pawlikowski, Michelle G. Craske, Georg W. Alpers

**Affiliations:** ^1^Chair of Clinical and Biological Psychology and Psychotherapy, Department of Psychology, School of Social Sciences, University of MannheimMannheim, Germany; ^2^Anxiety Disorders Research Center, University of CaliforniaLos Angeles, CA, USA; ^3^General Psychology: Cognition, Department for Informatics and Applied Cognitive Science, University of Duisburg-EssenEssen, Germany

**Keywords:** decision making, behavioral avoidance, social anxiety, facial expressions, psychopathology

## Abstract

Recent research indicates that angry facial expressions are preferentially processed and may facilitate automatic avoidance response, especially in socially anxious individuals. However, few studies have examined whether this bias also expresses itself in more complex cognitive processes and behavior such as decision making. We recently introduced a variation of the Iowa Gambling Task which allowed us to document the influence of task-irrelevant emotional cues on rational decision making. The present study used a modified gambling task to investigate the impact of angry facial expressions on decision making in 38 individuals with a wide range of social anxiety. Participants were to find out which choices were (dis-) advantageous to maximize overall gain. To create a decision conflict between approach of reward and avoidance of fear-relevant angry faces, advantageous choices were associated with angry facial expressions, whereas disadvantageous choices were associated with happy facial expressions. Results indicated that higher social avoidance predicted less advantageous decisions in the beginning of the task, i.e., when contingencies were still uncertain. Interactions with specific skin conductance responses further clarified that this initial avoidance only occurred in combination with elevated responses before choosing an angry facial expressions. In addition, an interaction between high trait anxiety and elevated responses to early losses predicted faster learning of an advantageous strategy. These effects were independent of intelligence, general risky decision-making, self-reported state anxiety, and depression. Thus, socially avoidant individuals who respond emotionally to angry facial expressions are more likely to show avoidance of these faces under uncertainty. This novel laboratory paradigm may be an appropriate analog for central features of social anxiety.

## INTRODUCTION

Avoidance is the characteristic action tendency associated with anxiety (Hofmann et al., 2009). It is triggered by emotional responses toward specific fear-relevant stimuli and can protect these emotional responses from extinction (Lovibond et al., 2009). Adaptive behavior, however, requires the individual to obtain reward or positive consequences. Investigating avoidance by itself, therefore, only accounts for one side of a two-sided balance between avoidance and approach (Stein and Paulus, 2009). This broader view on avoidance takes into account that anxious individuals miss out on potential benefits and therefore suffer costs. In social anxiety, these two tendencies are often in conflict with each other, because socially anxious individuals are explicitly aware of lost benefits, even for their most feared situations (Kashdan et al., 2008). For example, socially anxious individuals are often afraid of job interviews, although they are aware of the potential benefits for their career. Thus, they are in a conflict of opposing choices; to approach these benefits or avoid the situation to reduce anxiety. Pathological avoidance is, therefore, indicated by a dysfunctional shift toward avoidant decisions (Stein and Paulus, 2009). This shift results in the loss of benefits, which illustrates the impairments of patients with anxiety disorders. Studies investigating avoidance in the context of an approach–avoidance conflict in anxious individuals should, therefore, account for both fear-relevant as well as reward-related stimuli and consequences.

We recently combined these features in a novel experimental paradigm to investigate behavioral avoidance as a decision-making process (Pittig et al., 2014a). The paradigm was based on the Iowa gambling task (Bechara et al., 1994, 2000). In our modified gambling task, spider fearful participants continuously had to make decisions with the goal to maximize overall gains. Advantageous choices to obtain this goal were, however, associated with fear-relevant stimuli (i.e., pictures of spiders), such that avoidance of the fear-relevant stimuli resulted in the loss of long-term gains. In comparison to non-fearful participants, spider fearful participants consistently avoided the fear-relevant stimuli, despite the fact that these avoidant decisions resulted in overall cost in task performance. In addition, such avoidant decisions can result not only from specific fear-relevant stimuli, such as spiders, but also from novel fear conditioning experience, especially in anxious individuals (Pittig et al., 2014b). Thus, these laboratory experiments show that the presence of fear-relevant stimuli can trigger avoidant decisions in fearful individuals. As approach–avoidance conflicts are particularly relevant to social anxiety, we hypothesize that a similar bias may be observed in socially anxious individuals when confronted with stimuli specifically relevant to social interactions.

Facial expressions are one of the most important stimuli in social situations and determine the individual’s social behaviors. Emotional facial expressions are processed preferentially in the visual system (e.g., Alpers and Gerdes, 2007; Bublatzky et al., 2014b) and result in specific behavioral responses (Eisenbarth et al., 2011; Gerdes et al., 2012; Neumann et al., 2014). Angry facial expression are biologically rooted signals of threat (Öhman, 1986) and are specifically fear-relevant in subclinical and clinical levels of social anxiety. In support, recent research has pointed to a preferential processing of angry faces in healthy individuals (Mogg and Bradley, 1999; Fox et al., 2000). This preferential processing is pronounced in socially anxious individuals (Gilboa-Schechtman et al., 1999; Mogg et al., 2004; Klumpp and Amir, 2009; Wieser et al., 2009), which is accompanied by elevated amygdala activity in patients with social anxiety disorder (SAD; Stein et al., 2002). In addition, facial expressions can influence approach or avoidance tendencies. In this regard, approach-related motor responses are faster for happy faces, whereas avoidance-related responses are facilitated by angry faces (Marsh et al., 2005; Seidel et al., 2010). The difference in approach toward happy and angry faces is also evident in whole-body movements (Stins et al., 2011). The effect of a stronger avoidance response to angry faces may be elevated in socially anxious individuals, although a similar tendency was found for happy faces (Heuer et al., 2007). Finally, recent findings suggest that the difference in approach or avoidance tendencies is most pronounced when comparing angry to happy faces (Horstmann, 2003; Marsh et al., 2005; Seidel et al., 2010). Thus, confrontation with angry facial stimuli (compared to happy facial stimuli) may trigger avoidant decisions in socially anxious individuals.

There is indeed first evidence that angry facial expressions may generally bias rational decisions in healthy individuals (Averbeck and Duchaine, 2009; Furl et al., 2012). In these studies, the participants’ task was to find out whether selecting either a happy or an angry facial expression yielded more frequent reward. In general, decisions were biased toward selecting the happy face, even if prior evidence favored the angry facial expressions as advantageous choice (Averbeck and Duchaine, 2009). This effect of angry facial expressions on rational decisions may be more pronounced in socially anxious individuals. However, levels of social anxiety were not controlled in previous studies. To address this issue in order to evaluate whether repeated decisions are altered by subclinical levels of social anxiety and a tendency for social avoidance, the present study used a gambling task in which advantageous decisions were linked to angry facial expressions (similar to Pittig et al., 2014a,b).

In the field of decision making, several theories have stressed a general impact of emotional experience on decisions (Bechara et al., 1997; Loewenstein et al., 2001). These theories provide a powerful framework to investigate potential predictors of avoidant decisions in socially anxious individuals. For example, somatic marker theory (Damasio et al., 1991) suggests that emotional responses which are based on previous experience and activated during decision making can alter subsequent decisions. These responses are seen as embodied markers (or so called “gut-feelings”) which are linked to specific choices. Recent research on somatic markers using the Iowa gambling task or related paradigms typically investigated skin conductance responses (SCRs) as correlates and predictors of decisions (Bechara et al., 1997; Suzuki et al., 2003; Lawrence et al., 2006; Starcke et al., 2009; Pittig et al., 2014b). Importantly, the processing of facial expressions, especially angry expressions, is also related to physiological responses (Johnsen et al., 1995; Stein et al., 2002; Springer et al., 2007; Anokhin and Golosheykin, 2010; for an overview see Alpers et al., 2011). Thus, if angry facial expressions are presented during the consideration of different options, emotional responses to these faces may bias subsequent decisions, especially in socially anxious individuals. Since emotional responses to fear-relevant stimuli habituate with repeated presentation (Bradley et al., 1993; Bublatzky et al., 2014a), a potential bias on decisions should also be most pronounced for initial presentations. However, only one study so far reported that elevated SCRs can generalize form fear conditioning and subsequently predict more pronounced avoidant decisions (Pittig et al., 2014b). To this respect, the present study used SCRs as indicators of emotional arousal during social challenges (Schulz et al., 2008) and especially during decision-making (see Bechara et al., 1997; Adolphs, 2002).

## MATERIALS AND METHODS

### PARTICIPANTS

Thirty-eight students at UCLA participated for partial course credit. Exclusion criteria were assessed through self-report screening before the assessment and included any serious medical conditions, substance abuse/dependence, current/history of bipolar disorder, psychosis, organic/traumatic brain damage, and current use of psychotropic medications or medications that may influence autonomic state. All participants provided written informed consent. All procedures were approved by the UCLA Internal Review Board. Demographic, questionnaire, and neuropsychological data of the sample are shown in **Table 1**.

**Table 1:** Demographical, clinical, and neuropsychological data of the sample.

	Mean	SD
*N* (female)	38 (24)	
Age	19.89	(1.71)
SPIN	18.55	(14.49)
LSAS – fear scale	22.66	(13.88)
LSAS – avoidance scale	19.61	(13.63)
STAI – trait	36.61	(11.25)
STAI – state	30.47	(7.32)
BDI	5.28	(4.31)
GDT	6.26	(8.75)
IQ (based on LPS-4)	125.00	(12.10)

Means (and SD) for demographic, questionnaire, and neuropsychological data of the whole sample. *N,* number of participants; SPIN, social phobia inventory (Connor et al., 2000); LSAS, Liebowitz social anxiety scale (Fresco et al., 2001); STAI, state-trait anxiety inventory (Spielberger et al., 1983); BDI, beck depression inventory (Beck et al., 1996); GDT, game of dice-task (Brand et al., 2005); LPS-4, performance test system – subtest 4 (Horn, 1983).

### PROCEDURES

After informed consent was given, electrodes for the physiological measures were attached. Subsequently, participants completed a questionnaire battery including the self-rating form of the Liebowitz Social Anxiety Scale (LSAS-SR; Fresco et al., 2001), the trait version of the State-Trait Anxiety Inventory (STAI-T; Spielberger et al., 1983), and the Beck Depression Inventory (BDI-II; Beck et al., 1996). The LSAS is a 24-item questionnaire, commonly used to assess anxiety and avoidance in social interaction and performance situations. It has shown very good internal consistency, as well as good convergent and divergent validity (Heimberg et al., 1999). Participants rate each item twice, in terms of level of fear or anxiety (0 = “none”; 3 = “severe”; LSAS-Anxiety), and frequency of avoidance (0 = “never”; 3 = “usually”; LSAS-Avoidance). The social anxiety and avoidance scales served as main predictors in the present study.

Trait anxiety has been shown to influence decision making in the original Iowa gambling task. Although results were mixed (Miu et al., 2008; Werner et al., 2009), these studies demonstrate the need to control for general levels of unspecific trait anxiety. This was done with the 20-item STAI-T. The BDI is a self-report inventory which contains 21 items to measure the severity of depression. The BDI was used as a control measure, because depression symptoms may be associated with altered processing of reward (Mineka et al., 1998; Eshel and Roiser, 2010).

After a subsequent 5 min quiet sitting baseline, participants indicated their current state anxiety by completing the state version of the STAI. Afterward, they completed the gambling task, followed by the additional neuropsychological tasks. As the gambling task was always the first task to be completed, it was not influenced by the other tasks.

#### Social anxiety gambling task

**FIGURE 1:**
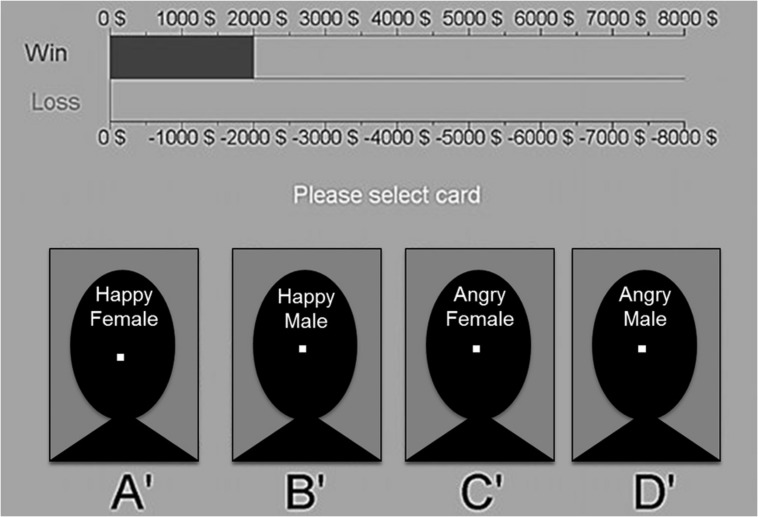
**Screenshot of the social anxiety gambling task with placeholders.** Disadvantageous decks **(A′,B′)** are depicting happy facial expressions, whereas advantageous decks **(C′,D′)** depict angry facial expressions. White squares in the middle of each deck represent the mouse-sensitive area where participants had to click to make a selection.

The gambling task was used to measure the impact of presentation of angry facial expressions on decision making. It was modeled after the Iowa gambling task (Bechara et al., 1994, 2000), see the screenshot in **Figure 1**. The task comprised four decks of cards (A, B, C, and D). Card backs depicted pictures of two happy facial expressions (decks A and B) and two angry facial expressions (decks C and D; all pictures approximately 9.15^∘^ × 5.73^∘^ visual angle). The pictures were taken from the Karolinska Directed Emotional Faces (KDEF; Lundqvist et al., 1998), a well validated picture set with moderately expressive facial expressions (Adolph and Alpers, 2010). Due to copyright terms of the KDEF, placeholders are used in **Figure 1**. Referring to the KDEF database the following pictures were displayed on the specific decks: deck A = AF01HAS, deck B = AM01HAS, deck C = AF21ANS, deck D = AM10ANS. Participants had to select one card at a time from one of the decks in a total of 100 trials. The mouse sensitive area for selecting a deck was reduced to a small square in the middle of each deck (approximately 0.57^∘^ visual angle), so that selecting a card required a fixation of the corresponding picture. After each trial, the mouse pointer moved back to the middle of the screen and had to be moved to the square again. Thus, each selection always required the participant to look at the corresponding deck and the depicted facial expression. After each selection a transparent gray shading of the decks was used to visualize this selection. This transparency ensured that the facial expressions were still visible, so that the selection could not be used as an avoidance strategy.

In order to compare physiological responses in each trial, there was another difference compared to the original Iowa gambling task. After each choice participants received feedback if they won or lost a specific amount of virtual money. In the original Iowa gambling task, gains and losses could occur simultaneously in one trial (Bechara et al., 1994, 2000). In contrast, in the present study participants could either win or lose on each trial to maintain a consistent single feedback after each trial.

Overall, decks A and B yielded large immediate gains, but occasionally even larger losses, resulting in long-term loss (-250 $ per 10 selections), and are considered disadvantageous choices for the goal of maximizing overall gain. The disadvantageous decks always depicted the two happy facial expressions. In contrast, decks C and D yielded small immediate gains, but also small occasional losses. Therefore, they resulted in long-term gains (+250 $ per 10 selections) and are considered advantageous choices. The advantageous decks always depicted the two angry facial expressions. Thus, to choose advantageously during the task, participants had to select the pictures of the angry facial expressions. As we focused on simulating the approach–avoidance conflict in individuals with elevated levels of social anxiety, we only investigated the link between advantageous choices and angry faces.

Participants were instructed to freely choose between the four decks with the goal to maximize their virtual monetary gains. In the beginning, they were not aware of the contingencies for gains and losses or the duration of the task. Hence, they had to use continuous feedback of gains and losses in order to learn which decks were advantageous or disadvantageous. Each participant started with a positive balance of $2000 and could continue to play even if they lost the entire starting amount. Analogous to analyses of the Iowa gambling task (e.g., Bechara et al., 2000), the 100 trials of the gambling task were analyzed in five blocks of 20 trials each. The number of advantageous choices from decks C and D was used as the dependent variable. Higher scores indicate a higher overall outcome in the task due to choosing advantageous decks with angry facial expressions.

After completing the task, participants were asked to rate the pictures of each facial expression. All ratings were given on a 10-point Likert scale for valence/pleasantness (0 = “very unpleasant”; 9 = “very pleasant”) and arousal (0 = “not at all aroused”; 9 = “extremely aroused”).

#### Neuropsychological control measures

Risky decision making: game of dice task. The game of dice task (GDT; Brand et al., 2005) is a computerized dice task and was administered to control for general differences in risky decision making independent of fear-relevant stimuli. A virtual dice is thrown and participants are asked to maximize a fictitious starting capital by guessing the correct number thrown in 18 trials. Participants can guess a single number or two, three, or four numbers together. If the guess matches the thrown number, participants win a specific amount of virtual money. If not, they lose the same amount. Non-risky choices have a winning probability of 50% or higher and are linked to lower gains (i.e., a combination of three numbers with a 50% probability to win 200 € and a combination of four numbers with a 66.67% probability to win 100 € ). Risky choices have a lower winning probability, but are linked to higher gains (i.e., a single number with 16.67% probability to win 1000 € or a combination of two numbers with a 33.33% probability to win 500 € ). For analysis, a net score was calculated by subtracting the number of risky choices from the number of safe choices. Thus, a higher net score indicates more non-risky choices.

Logical reasoning: performance test. Potential differences in reasoning abilities were controlled using the Performance test system – Subtest 4 (LPS-4; Horn, 1983). This was done because such differences can influence decision making in the Iowa gambling task (Bechara et al., 1997). The LPS-4 is a non-verbal test used to estimate logical reasoning. Participants have 8 min to find a single error in a logical order of letters and numbers in a total of 40 rows. The number of errors correctly identified can be used to estimate the logical reasoning skills and intelligence of the participant.

### PHYSIOLOGICAL ASSESSMENT

Electrodermal activity (EDA) was continuously recorded as measure of emotional responses during decision making using BIOPAC instrumentation (MP150 Data Acquisition System for Windows; BIOPAC Systems, Inc.). Data monitoring, acquisition, and analysis were conducted with AcqKnowledge software (AcqKnowledge 4.1; BIOPAC Systems, Inc.). One disposable Ag/AgCl electrode on the left clavicle served as ground electrode. EDA was recorded using BIOPAC skin conductance instrumentation with a constant voltage of 0.5 V (sampling rate = 62.50 Hz). Two disposable Ag/AgCl electrodes with electrodermal conducting gel were attached to the palmar surface of the middle phalanges of the second and third fingers of the non-dominant hand. Participants were instructed to avoid larger movement as to not bias the physiological responses. Data recording was monitored online and artifacts (e.g., movement, sneezing, etc.) were recorded by a research assistant who observed the assessment from an adjacent room. All sections with such events were removed from further analysis.

#### Skin conductance responses

Each choice during the gambling task was flagged by a digital marker. SCRs were analyzed for two intervals (see Bechara et al., 1997; Starcke et al., 2009): feedback SCRs were analyzed in the 5 s following each choice and anticipatory SCRs were analyzed 5 s before every choice. Six second intervals between two consecutive choices allowed sufficient time to score both types of SCRs without serious overlaps (Bechara et al., 1999; Starcke et al., 2009). The mean interval time between two consecutive choices in the present study was 10.71 s (SD = 0.93).

EDA data were filtered with a digital low-pass 2 Hz FIR filter. A 0.05 Hz FIR high-pass filter was used to obtain phasic SCRs. SCRs were calculated as the maximum increase in skin conductance during 5 s before and after each choice. A threshold of 0.02 Mirco-Siemens (μS) was used; all SCRs below this threshold were scored as zero (zero responses were included in the calculation of mean responses). For range correction, SCRs were divided by the largest SCR of each participant (SCR corrected = SCR raw/SCR maximum; Lykken and Venables, 1971) and the square root was taken to obtain normal distribution (Dawson et al., 2007). Skin conductance recordings could not be analyzed for two participants due to equipment failure.

The present analyses focused on SCRs during the first block of the gambling task in order to predict subsequent choices, because the impact of SCRs was expected to be strongest in initial trials. SCRs of the first block were analyzed in six different SCR categories (see Bechara et al., 1999; Starcke et al., 2009); two anticipatory SCR categories (before choosing an advantageous vs. disadvantageous deck) and four feedback SCR categories that were subdivided by deck (after choosing an advantageous vs. disadvantageous deck) and outcome (win vs. loss).

### STATISTICAL ANALYSES

All questionnaire data were examined for outliers (defined as values >2.5 SD from the mean) and for normal distribution. Detected outliers (0.19% of all data) were replaced with the closest, non-outlier value (Winsor method; see Guttman, 1973). Hierarchical linear growth curve models (HLMs with random intercept and random slope) were built with gambling task scores as the dependent variable to model repeated decisions (level-1) nested in different individuals (level-2) using HLM 6.08 software (Raudenbush and Bryk, 2002; Raudenbush et al., 2004). HLM is particularly well-suited for the analysis of repeated data, because it does not require independence of observations for repeated observations and produces lower Type I error rates than standard GLM procedures (Raudenbush and Bryk, 2002).

Examination of the raw data and a non-significant result for quadratic change over blocks resulted in the use of linear level-1 components to model gambling task scores (i.e., changes across blocks were modeled in a linear way). Both intercept and slope for the unconditional linear model showed significant variability. Therefore, gambling task blocks (block 1–5) were entered as the repeated level-1 predictor. On level-2, the different clinical and neuropsychological variables were entered to test if these variables predicted gambling task scores for the first block (intercept) and linear change across blocks (slope). All variables were mean-centered before being entered into the model and before interaction terms were calculated to reduce multicollinearity. For interaction analyses, multiplication terms were entered together with their corresponding main effect variables. To investigate the impact of social anxiety and avoidance on decision making, the effects of self-reported social anxiety (LSAS-Anxiety) and social avoidance (LSAS-Avoidance) were tested on level-2. Further, effects of the additional control variables were tested in a similar way, including trait anxiety (STAI-T), state anxiety (STAI-State), depression (BDI), intelligence (LPS-4), and general risky decision making (GDT). Before building a combined model including multiple predictors, each predictor was separately tested on level-2 to ensure that a potential effect was not covered by the effect of another variable or poor statistical power. Afterward, significant predictors were combined in one model to test for incremental predictive effects. In addition, potential incremental effects of the different SCRs generated during the first block were investigated by entering SCRs of the six categories on level-2. To this end, interaction terms between anxiety or social avoidance and the different SCRs as well as main effects of the SCRs were entered into the model.

Tests of HLM assumptions did not yield serious violations. Assumptions were tested using the Kolmogorov–Smirnov test for normal distribution of level-1 and level-2 residuals, a χ^2^ test for homogeneity of level-1 residual variance, and visual inspection of scatterplots. Reported *R*^2^ effect sizes represent the proportion of variance explained by adding the level-2 variables; these effect sizes were calculated by subtracting the variance obtained with the level-2 predictors from the variance obtained without the level-2 predictor, and dividing by the latter (Raudenbush and Bryk, 2002). *R*^2^ values were separately calculated for both HLM intercept and linear slope. In addition, the level-1-only model (without any level-2 predictors) served as a reference model to evaluate if the observed data were better explained by adding the level-2 variables to the model. Therefore, a χ^2^ variance-covariance components based likelihood test was used (see Raudenbush and Bryk, 2002).

## RESULTS

### GAMBLING TASK PERFORMANCE

**FIGURE 2:**
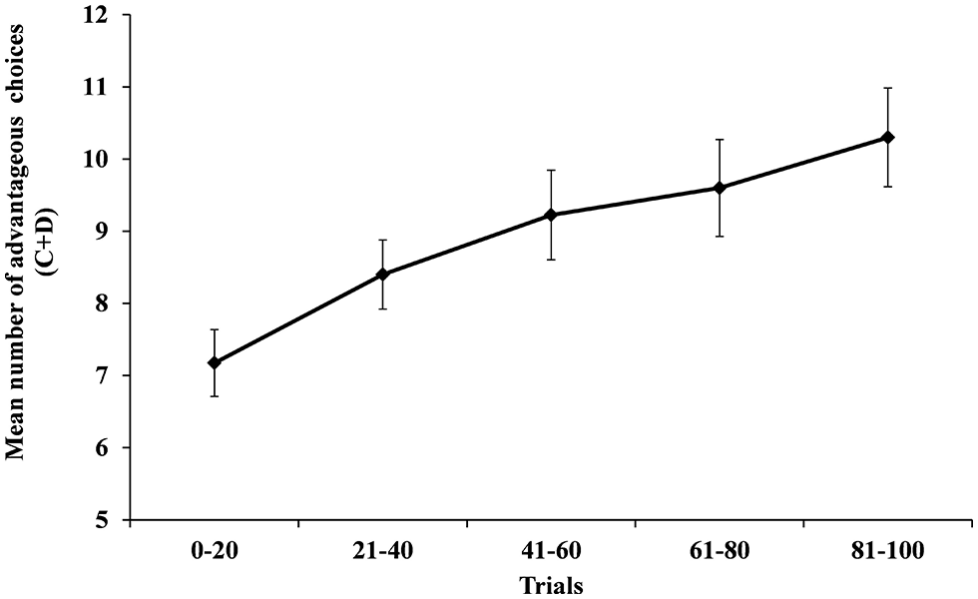
**Means (with SE) of the number of choices from deck C + D.** Higher scores indicate more frequent choices from the advantageous decks depicting angry facial expressions.

Means and SD of choices from the advantageous decks for the five blocks of the gambling task yielded sufficient variance for regression analyses, see **Figure 2** (block 1: *M* = 7.18, SD = 2.30; block 2: *M* = 8.40, SD = 3.03; block 3: *M* = 9.23, SD = 4.56; block 4: *M* = 9.60, SD = 4.24; block 5: *M* = 10.30, SD = 4.32). The level-1-only model including blocks (no level-2 predictors) yielded significantly fewer advantageous choices with angry facial expressions in block 1, *B* = 7.42, SE = 0.35, *t*_(36)_ = -7.30, *p* < 0.001, and a significant linear increase across blocks, *B* = 0.64, SE = 0.16, *t*_(36)_ = 3.96, *p* < 0.001. So, all participants combined made fewer advantageous choices with angry facial expressions at the beginning and successively learned to make more advantageous choices. Learning was also evident in the assessment of individual contingency awareness ^1^. However, at the end of the task all participants combined did not show a clear preference toward the advantageous deck, as indicated by **Figure 2**.

### EFFECT OF SOCIAL ANXIETY AND SOCIAL AVOIDANCE ON DECISION MAKING

In the HLM with social avoidance as only level-2 predictor, higher social avoidance predicted fewer advantageous choices from the decks with angry facial expressions in block 1 (intercept), *B* = -0.06, SE = 0.02, *t*_(36)_ = -3.64, *p* = 0.001. In the HLM with social anxiety as only level-2 predictor, higher social anxiety yielded a similar effect, but with a smaller effect size, *B* = -0.05, SE = 0.02, *t*_(36)_ = -2.31, *p* = 0.027. In both HLMs, no effect was found for change across blocks for social anxiety, *B* = 0.01, SE = 0.02, *t*_(36)_ = 0.70, *p* = 0.489, or social avoidance, *B* = 0.01, SE = 0.01, *t*_(36)_ = 0.88, *p* = 0.386.

### EFFECT OF CONTROL VARIABLES ON DECISION MAKING

For all following results the specific control variable was entered as single level-2 predictor into an HLM with blocks entered as repeated level-1 factor. In the HLM with trait anxiety as only level-2 predictor, higher trait anxiety predicted fewer advantageous choices from the decks with angry facial expressions in block 1 (intercept), *B* = -0.07, SE = 0.03, *t*_(36)_ = -2.84, *p* = 0.008, but had no effect on change across blocks, *B* = -0.01, SE = 0.02, *t*_(36)_ = -0.62, *p* = 0.538. In addition, when depression scores were entered alone on level-2, higher depression scores also predicted fewer advantageous choices in block 1, *B* = -0.18, SE = 0.08, *t*_(36)_ = -2.17, *p* = 0.036, but did not show a significant effect on change across blocks, *B* = 0.01, SE = 0.03, *t*_(36)_ = 0.35, *p* = 0.732.

For all other HLMs with a single control variable as predictor, the effects on intercept and slope were not significant: (1) age: intercept: *B* = 0.03, SE = 0.21, *t*_(36)_ = 0.13, *p* = 0.898, slope: *B* = -0.13, SE = 0.10, *t*_(36)_ = -1.40, *p* = 0.171; (2) sex: intercept: *B* = -0.45, SE = 0.69, *t*_(36)_ = -0.66, *p* = 0.517, slope: *B* = -0.21, SE = 0.39, *t*_(36)_ = -0.54, *p* = 0.591; (3) state anxiety: intercept: *B* = -0.08, SE = 0.05, *t*_(36)_ = -1.68, *p* = 0.101, slope: *B* < 0.01, SE = 0.02, *t*_(36)_ = 0.07, *p* = 0.946; (4) GDT: intercept: *B* = 0.01, SE = 0.04, *t*_(36)_ = 0.23, *p* = 0.819, Slope: *B* = 0.02, SE = 0.02, *t*_(36)_ = 0.73, *p* = 0.468; (5) Estimated IQ (LPS-4): intercept: *B* = -0.02, SE = 0.03, *t*_(36)_ = -0.57, *p* = 0.572, Slope: *B* < 0.01, SE = 0.02, *t*_(36)_ = 0.50, *p* = 0.622.

Next level-2 predictors which yielded significant effects when entered as single level-2 predictor were combined in one model to test incremental predictive power. In the model including social avoidance and anxiety, trait anxiety, and depression scores, only social avoidance, *B* = -0.04, SE = 0.01, *t*_(35)_ = -2.39, *p* = 0.023, and trait anxiety, *B* = -0.05, SE = 0.02, *t*_(35)_ = -2.20, *p* = 0.034, incrementally predicted fewer advantageous choices from the decks with angry facial expressions in block 1. In the combined model, no significant effect was found for social anxiety, *B* = -0.03, SE = 0.03, *t*_(35)_ = 1.10, *p* = 0.281, or depression, *B* = -0.08, SE = 0.12, *t*_(34)_ = -0.70, *p* = 0.488. In addition, no variable predicted linear change across blocks in the combined model; social avoidance, *B* = 0.01, SE = 0.01, *t*_(35)_ = 1.75, *p* = 0.089, social anxiety, *B* = 0.02, SE = 0.02, *t*_(35)_ = 1.24, *p* = 0.225, trait anxiety, *B* = -0.02, SE = 0.02, *t*_(35)_ = -1.26, *p* = 0.217. Thus, social anxiety and depression scores were dropped from the model. The inclusion of social avoidance and trait anxiety on level-2 resulted in significantly less model deviance of observed from estimated data compared to the level-1-only model, χ^2^= 8.98, *p* = 0.003. In summary, after analysis of the additional variables, social avoidance and trait anxiety both yielded significant beta weights and were the only variables showing incremental predictive values in a model with more than just one predictor.

### EFFECTS OF TRAIT ANXIETY AND SOCIAL AVOIDANCE ON SUBSEQUENT BLOCKS

To further investigate if the effects of self-reported social avoidance and trait anxiety were consistent throughout the task, the intercept analyses were repeated for block 2–5. For STAI-T, the effects were significant for all subsequent blocks; block 2, intercept: *B* = -0.07, SE = 0.02, *t*_(35)_ = -3.28, *p* = 0.003; block 3, intercept: *B* = -0.09, SE = 0.03, *t*_(35)_ = -2.99, *p* = 0.006; block 4, intercept: *B* = -0.11, SE = 0.04, *t*_(35)_ = -2.54, *p* = 0.016; and block 5, intercept: *B* = -0.13, SE = 0.06, *t*_(35)_ = -2.24, *p* = 0.032. In contrast, the effects for LSAS-Avoidance were not significant beyond block 1; block 2, intercept: *B* = -0.02, SE = 0.02, *t*_(35)_ = -1.11, *p* = 0.277; block 3, intercept: *B* = -0.01, SE = 0.03, *t*_(35)_ = -0.17, *p* = 0.870; block 4, intercept: *B* = 0.01, SE = 0.04, *t*_(35)_ = 0.32, *p* = 0.750; and block 5, intercept: *B* = 0.03, SE = 0.05, *t*_(35)_ = 0.59, *p* = 0.560. Thus, self-reported trait anxiety consistently predicted fewer advantageous choices from the decks with angry facial expressions throughout the task, whereas social avoidance had an impact only in the first block.

### INTERACTIONS WITH EMOTIONAL RESPONSES DURING DECISION MAKING

#### Initial decisions: social avoidance and SCRs

**FIGURE 3:**
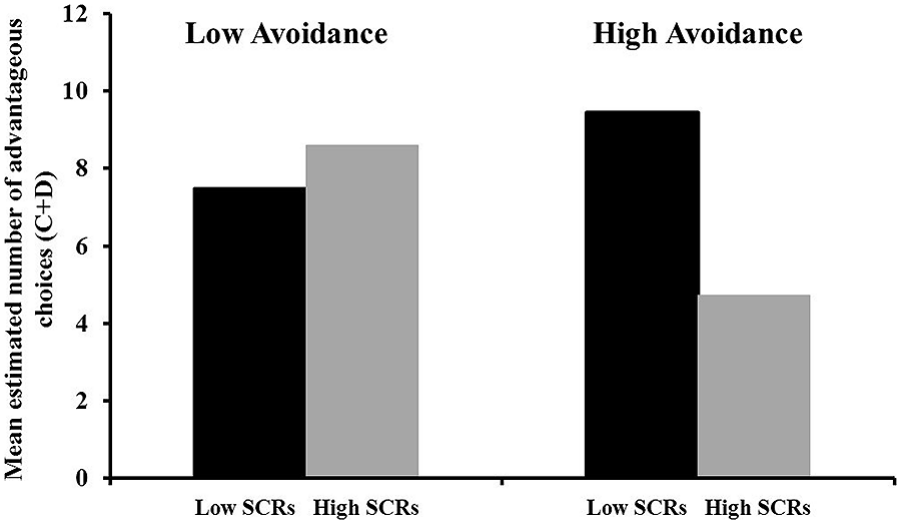
**Interaction illustration by HLM estimated number of choices from deck C+D in the first block of the social anxiety gambling task for high vs. low social avoidance with high vs. low skin conductance responses (SCRs) before choosing an advantageous deck with angry facial expressions.** Mean scores of the upper and lower quartile were used to illustrate the interaction effect. Higher scores indicate more frequent choices from the advantageous decks depicting angry facial expressions.

In order to evaluate the influence of emotional responses during decision making, the different SCR categories and their interaction with social avoidance and trait anxiety were analyzed in different HLMs (each included the main effect of the specific SCR category, social avoidance and trait anxiety, and the interaction terms). For intercept analyses, no significant main effects on intercept were found for any of the SCR categories of block 1, all *t*s < 1.90, all *p*s > 0.08. In addition, no interaction effects on intercept were found between SCRs in block 1 and trait anxiety, all *t*s < 1.70, all *p*s > 0.10. However, analyses yielded a significant interaction of social avoidance and anticipatory SCRs before choosing an advantageous deck with angry facial expressions, *B* = -0.43, SE = 0.16, *t*_(32)_ = -2.62, *p* = 0.015. In order to illustrate the significant interaction, results were compared for different levels of the predictors (social avoidance and anticipatory SCRs before choosing an advantageous deck with angry facial expressions). Therefore, high and low scores for social avoidance and the anticipatory SCRs of the present sample were calculated by using mean scores for the upper and lower quartile. These scores for low and high levels were entered back into the regression and estimated results for high and low scores were plotted for visual interpretation of the interaction. **Figure 3** illustrates that in the first block, only high social avoidance in combination with higher anticipatory SCRs predicted less frequent choices of the advantageous decks with angry facial expressions. No further interactions between SCR categories and social avoidance were found, all *t*s < 0.65, all *p*s > 0.54.

#### Learning of advantageous decisions: trait anxiety and SCRs

**FIGURE 4:**
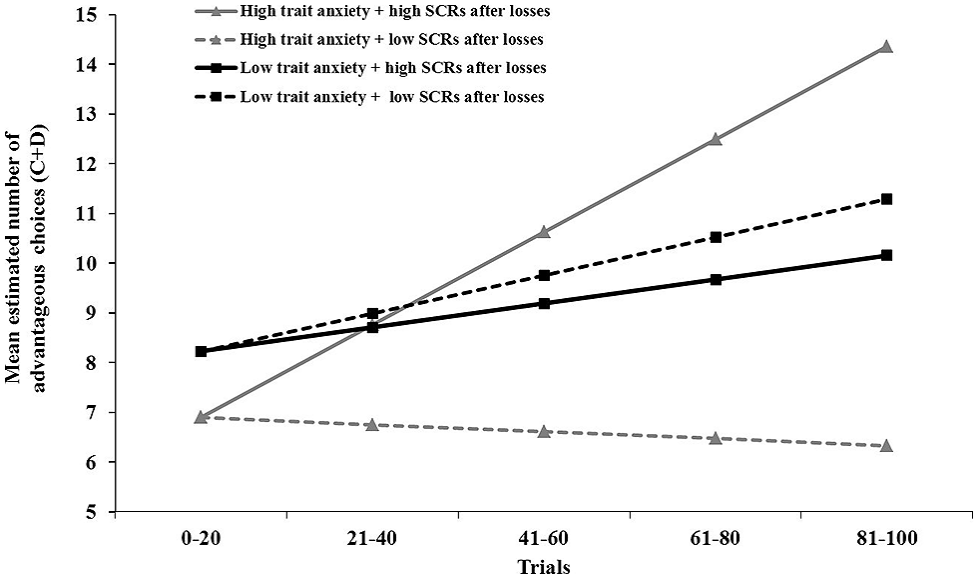
**Interaction illustration by HLM estimated number of choices from deck C+D for high vs. low self-reported trait anxiety with high vs. low skin conductance responses (SCRs) after loss feedbacks in the first block of the social anxiety gambling task.** Mean scores of the upper and lower quartile were used to illustrate the interaction effect. Higher scores indicate more frequent choices from the advantageous decks depicting angry facial expressions. Positive slopes indicate increased learning of advantageous choices.

Slope analyses were conducted with the same predictors as intercept analyses (main and interaction effect of social avoidance, trait anxiety, and the single SCR categories). No significant main effects on slope were found for any of the SCR categories, all *t*s < 1.00, all *p*s > 0.33. In addition, no interaction effects on slope were found for interactions between SCR categories and social avoidance, and most of the interaction effects between SCR categories and trait anxiety, all *t*s < 1.30, all *p*s > 0.21. However, the interaction between SCRs after loss feedbacks (for both advantageous and disadvantageous decks) and trait anxiety significantly predicted change across the task, *B* = 0.20, SE = 0.07, *t*_(32)_ = 2.77, *p* = 0.010. **Figure 4** illustrates that high trait anxiety combined with lower SCRs after losses resulted in decreased learning. However, a steeper learning curve occurred in high trait anxious participants, if participants showed higher SCRs to losses in the initial block of the task.

Finally, only the two significant interaction terms (social avoidance and anticipatory SCRs before choosing an advantageous deck for intercept; trait anxiety and SCRs after losses for slope) as well as the corresponding main effects were included into one model to test for incremental effects of the interaction terms. The inclusion yielded significantly less deviances of observed data from modeled data compared to the model with trait anxiety and social avoidance only, χ^2^= 13.46, *p* = 0.001. Compared to the level-1-only model, inclusion of the level-2 predictors explained additional variance of initial decisions in block 1 (*R*^2^ = 0.23) and change across blocks (*R*^2^ = 0.10).

### PICTURE RATINGS

After completion of the gambling task, both pictures with angry facial expressions (female angry face: *M* = 1.81; SD = 1.35; male angry face: *M* = 1.97; SD = 1.46) were rated as significantly less pleasant compared to the happy facial expressions (female happy face: *M* = 7.44; SD = 1.42; male happy face: *M* = 6.75; SD = 1.57), all *t*s > 12.16, all *p*s < 0.001. In addition, arousal ratings were significantly higher for the male angry face (*M* = 4.97; SD = 2.98) compared to both happy facial expressions (female happy face: *M* = 2.94; SD = 2.53; male happy face: *M* = 3.06; SD = 2.62), all *t*s > 3.30, all *p*s < 0.003, but not for the female angry facial expressions (female angry face: *M* = 3.69; SD = 2.92) compared to all happy facial expressions, all *t*s < 1.27, all *p*s > 0.21.

## DISCUSSION

The present study yielded evidence that task-irrelevant, but fear-relevant, angry faces exert a bias on choices in ambiguous situations, which is pronounced in individuals with elevated levels of social anxiety. This influence was observed in an experimental gambling task linking advantageous choices with angry facial expressions. The present findings were not explained by age, sex, state anxiety, logical reasoning, or risky decision making. Overall, all participants combined made fewer advantageous choices with angry facial expressions at the beginning. Furthermore, participants successively learned to make more advantageous choices, but did not exhibit a clear preference for the advantageous decks at the end.

These findings may suggest a more universal bias of angry facial expressions on decision making which is not limited to elevated levels of social anxiety and avoidance. Indeed, previous studies reported a similar initial bias of angry facial expressions on rational choices for healthy participants (Averbeck and Duchaine, 2009). However, in this previous study the bias was limited to early trials and participants integrated feedback information to change their decisions. In the present study, this correction to initial decisions was also evident in explicit contingency ratings and increasing advantageous choices across the task. However, across all participants no clear preference for the advantageous choices was found by the end of the task. The latter results are not in line with most findings of healthy participants completing the original Iowa Gambling Task (e.g., Bechara et al., 1997, 1999, 2000; Brand et al., 2007). There may be different explanations for this mismatch of contingency awareness and persisting behavioral preference toward happy facial expressions at the end of the task.

In line with the finding of a universal bias, angry facial expressions may have exerted a more sustained bias on decision making behavior than on cognitive evaluation (contingency awareness) in our study. This sustained bias would have led to fewer selections of angry facial expressions despite developing knowledge of an advantageous outcome of these decks (for examples of discrepant evaluation and behavior see Strack and Deutsch, 2014). Alternatively, it is not clear when precisely participants acquired awareness of contingencies, because explicit awareness was only assessed after completion of the task. Due to the limited experience with the advantageous decks at the beginning, it may be possible that participants became explicitly aware only during the last blocks of the task. Whereas this late awareness can be seen in explicit knowledge after the task, it would be too late to result in a significant preference of advantageous choices. To further clarify the mismatch of contingency awareness and a behavioral preference, future research may, therefore, incorporate online contingency and expectancy ratings throughout the task (e.g., see Maia and McClelland, 2004).

### INITIAL CHOICES, SOCIAL AVOIDANCE, AND EMOTIONAL RESPONSES

Across all participants, the decks with angry facial expressions were avoided initially. As hypothesized, these avoidant decisions were more pronounced in participants with a higher level of social avoidance. This effect of social avoidance was, however, limited to the beginning of the task when contingencies were still obscure and vanquished at the end of the task. These results are in line with recent findings of a bias on initial decisions in healthy participants (Averbeck and Duchaine, 2009). In this study, healthy participants also showed initial avoidance of angry compared to happy facial expressions, which diminished in later trials. In the present study, this initial avoidance of angry facial expressions was elevated in individuals with elevated levels of social anxiety. These present results further support recent studies that found similar avoidant decisions in individuals with fear of spiders (Pittig et al., 2014a) and in healthy participants after fear conditioning experience (Pittig et al., 2014b). Thus, avoidant decision making under uncertainty seems to be common across different types of fear and anxiety. Detailed understanding of the underlying mechanisms of development and reduction of avoidant decisions may inform behavioral treatments which target pathological avoidance behaviors.

For initial choices, a significant interaction was found between social avoidance and anticipatory SCRs before choosing an advantageous deck with angry facial expressions. Only high social avoidance in combination with higher anticipatory SCRs resulted in fewer choices of angry facial expressions. This interaction provides evidence that avoidance is not triggered by angry facial expressions for every individual with high levels of social avoidance, but rather depends on congruent elevated emotional responses. As social anxiety represents a multidimensional construct with multiple stimuli and cognitive processes as potential triggers for anxious responses (Hofmann et al., 2004; Bögels et al., 2010), it is likely that angry facial expressions were not fear-relevant stimuli for all participants with elevated levels of social anxiety. This may have resulted in lower emotional responses and subsequently in missing avoidant decisions for some participants.

As SCRs are sensitive to many cognitive and affective processes, it is difficult to interpret the specific interaction effects without doubt. Previous research suggested different explanations for anticipatory SCRs in the Iowa gambling task, for example, growing awareness of contingencies or reward and punishment expectancies (for a review see Dunn et al., 2006). Previous studies, however, did not find any specific anticipatory reactions in the initial block of the task (Bechara et al., 1997). In addition, the present results indicate that the interaction of social avoidance and emotional responses was specific for decks with angry facial expressions. Findings, thus, may favor the interpretation that the initial bias on decisions was related to the angry facial expressions. It seems that early responses to the angry facial expressions may have been the most prominent information for initial decisions. Whereas the facial expressions can be identified immediately, task-relevant information or emotional labels related to gains and losses tend to develop at a later stage (Bechara et al., 1997; Brand et al., 2007). Thus, fear-relevant information may have had the strongest impact under uncertainty due to missing explicit knowledge. With little knowledge about outcomes, individuals with a high tendency to avoid social stimuli and situations may have followed their default avoidance strategy if they experienced higher emotional responses.

However, pronounced avoidance in highly avoidant individuals was limited to initial trials of the gambling task, when the contingencies were not yet detected. In later trials, no effect of elevated social avoidance was found above and beyond the general tendency to choose fewer cards from the decks with angry facial expressions (advantageous decks) across all participants. Thus, pronounced avoidance was limited to trials with uncertainty about gain-related consequences, in which it is likely that no strong conflict between avoidance and approach was evident. After further experience with the contingencies, initial avoidance may have been overcome in the presence of an opposing reward-related goal. In the present subclinical sample, socially avoidant individuals did not engulf long-term costs. This findings differs from our previous findings in a sample of spider fearful participants, where avoidant decisions were more consistent (Pittig et al., 2014a). Possibly, these differences may be explained by the specificity of the fear-relevant stimuli. Whereas spiders are specific fear-relevant stimuli for spider-fearful individuals, multiple stimuli, and cognitive processes may trigger anxious responses in social anxiety, as mentioned above (Hofmann et al., 2004; Bögels et al., 2010).

Current decision-making theories further highlight the importance of emotional and cognitive influences in decision making (Bechara et al., 1997; Loewenstein et al., 2001). Changes in both processes could have influenced the decrease in the effect of avoidance over time. First, participants were exposed to the same pictures in all 100 trials. This could have caused a habituation of emotional responses to the angry facial expressions. Thus, initial avoidance may have decreased due to reduced aversiveness of the angry facial expressions, which were subsequently not strong enough to bias decisions persistently. In addition, research on the Iowa gambling task showed that uncertainty is especially pronounced during the initial block, whereas explicit knowledge starts to develop in block 2 or 3 (Brand et al., 2007). With more experience, more explicit knowledge about the benefits of selecting the angry facial expressions was acquired. This acquisition of explicit knowledge may have mitigated the effect of the angry facial expressions and fostered the approach tendency.

### LEARNING ACROSS THE TASK, TRAIT ANXIETY, AND EMOTIONAL RESPONSES TO LOSSES

First results suggested a general negative effect of trait anxiety on advantageous decision making. This effect was further clarified by a significant interaction between trait anxiety and SCRs after early losses. High trait anxiety combined with low SCRs was associated with impaired learning of advantageous decisions. Conversely, elevated learning of advantageous choices occurred in high trait anxiety in combination with higher SCRs to losses during initial decisions.

These findings can be linked to previous studies on the impact of high trait anxiety and SCRs on decision making in the original Iowa gambling task. First, prior studies also reported a link between feedback SCRs and decision making (Suzuki et al., 2003; Lawrence et al., 2006) and showed that participants with higher SCRs to losses performed better in the Iowa gambling task (Starcke et al., 2009). Regarding elevated levels of trait anxiety, previous results were mixed. Whereas some findings suggest a negative impact of high trait anxiety on decision making (Miu et al., 2008), others contrarily suggest a beneficial effect (Werner et al., 2009). Here, the latter findings supported a positive correlation between high trait anxiety and physiological responses to feedback. Combined, these findings may suggest that the effect of trait anxiety in the present study could be more closely linked to outcome-related features of the task than to the facial expressions. This may indicate that stronger emotional responses can augment advantageous decision making, if they are related to goal-relevant features of the task.

The impact of initial reaction to losses was not observed in participants with low trait anxiety. This specificity of early losses in high trait anxiety may be linked to a higher loss aversion in highly anxious individuals (see Hartley and Phelps, 2012). In addition, the immediate impact of emotional responses on decision making may be lower in low trait anxious individuals, whereas the use of cognitive strategies may be more pronounced. Such cognitive strategies depend on explicit knowledge, which starts to develop at a later stage of the task (Brand et al., 2007). In contrast to the use of cognitive strategies, high trait anxious individuals may be more engaged in emotional reasoning. Here, the impact of responses to early losses could be mediated by a higher perceived intensity of negative outcomes in high trait anxious individuals (Maner and Schmidt, 2006) and the tendency to use fewer cues in reasoning tasks (Leon and Revelle, 1985). Thus, if highly trait anxious individuals show elevated responses to goal-relevant features, they may be more prone to immediately direct future decisions following these information.

### LIMITATIONS AND FUTURE RESEARCH

There are some limitations to the present study which may be relevant for future research. First, we treated anxiety and depression as dimensions rather than nosological categories and participants were not selected for clinical levels of anxiety or depression. We expect that the effects observed here may be even be more pronounced in more severe degrees of anxiety an in patients with SAD. For example, individuals with clinical levels of social avoidance may show a more consistent pattern of avoidant decisions. However, a discussion of potential perspectives of social phobia warrants further examination in clinical populations. Future research may, therefore, replicate the present findings within a sample of patients with SAD. Similarly, the impact of clinical levels of depression may be tested. Results from our study in fear of spiders suggest that effects of fear-relevant stimuli on avoidant decisions may be similar in above-threshold individuals (Pittig et al., 2014a).

Second, whereas the present sample size provided enough power to detect effects of social avoidance and trait anxiety, it might not be large enough to detect small effects of the remaining variables. Future research may, for example, further investigate potential effects of gender, age, intelligence, or risky decision making in general. However, this was not the primary focus of the present study and controlling for these variables did not change the main results.

Third, exclusion criteria, especially the absence of medical or other psychiatric conditions were only assessed via self-report of the participants. No standardized clinical interviews were completed to more thoroughly rule out a history of psychiatric disorders. Although this might have resulted in a miss of specific or past psychiatric symptoms, the current sample mainly consisted of students. Nevertheless, future research may incorporate standardized interview to further examine the role of different or past psychiatric symptoms.

Fourth, awareness of reward contingencies was only assessed after completion of the task. This procedure was used to prevent a potential bias of repeated contingency ratings throughout the task on learning of an advantageous strategy. However, it remains unclear when participants developed awareness of contingencies and if they still continued to avoid the angry facial expressions afterward. In order to further clarify the relationship between explicit knowledge of reward contingencies and the effect of angry facial expressions, future research may incorporate online contingency and expectancy ratings throughout the task (e.g., see Maia and McClelland, 2004).

### CONCLUSION

In summary, angry facial expressions trigger avoidant decisions in individuals with elevated levels of social anxiety, but only in those who initially experience strong emotional responses toward these stimuli. Emotional responses can also be beneficial and increase advantageous decision making, if they are goal-relevant. This highlights the opposing impact of emotions on decision making and calls for the need to account for both types of emotional responses when investigating avoidance behavior in anxiety.

## Conflict of Interest Statement

The authors declare that the research was conducted in the absence of any commercial or financial relationships that could be construed as a potential conflict of interest.

## Footnotes

1 Participants were asked about their contingency knowledge after the task (see also Bechara et al., 1997; Maia and McClelland, 2004): (1) “Tell me all you know about what is going on in this game?”, (2) “Did you find any difference between the decks?”, (3) “Suppose you select 10 new card from deck A (B, C, D) will you on average win or lose money?”, (4) “If you have to choose only one deck, which one will you choose in order to maximize your long-term gain?”. Participants were judged as having explicitly learned the correct contingencies, if they indicated correct answers to question 3 and 4. Seventy four percent of the participants were judged to be explicitly aware of the contingencies. In addition, analyses that only include participants judged as aware of contingencies yielded the same pattern of results as analyses including the entire sample.
